# National variation in guidance for the management of pregnant women presenting with major trauma

**DOI:** 10.1308/rcsann.2024.0011

**Published:** 2024-04-02

**Authors:** C Demetriou, W Eardley, M-C Rebeiz, CB Hing

**Affiliations:** ^1^ East of England Deanery, UK; ^2^ South Tees Hospitals NHS Foundation Trust, UK; ^3^ St George’s University Hospitals NHS Foundation Trust, UK

**Keywords:** Major trauma, Pregnancy, Guideline

## Abstract

**Introduction:**

The initial assessment of pregnant women presenting with significant injuries is more complicated than that of non-pregnant women because of physiological and anatomical changes, and the presence of the fetus. The aim of this study was to determine whether guidelines for the early management of severely injured pregnant women exist, which aspects of assessment/management they cover and to what extent there is national consistency.

**Methods:**

A freedom of information request was submitted to 125 acute National Health Service trusts in England and six in Wales. The trusts were asked to confirm whether they have a guideline for the management of major trauma in pregnant women presenting to the emergency department and what the guidelines were.

**Results:**

In total, 96.2% of trusts responded, of which 19% have a specific guideline and 7.9% have a generic guideline for assessing pregnant women in the emergency department, irrespective of injury severity. Of the responding trusts, 19.8% have a protocol that specifies when an obstetric trauma call should be put out by the emergency department and when a pregnant woman should be transferred to a major trauma centre for definitive management. Our results found that 69.8% routinely call obstetrics or gynaecology to the trauma call compared with 36.5% calling paediatrics.

**Conclusions:**

The heterogeneity evident across trusts necessitates the establishment of national guidelines for the assessment of pregnant women with major trauma to standardise communication and delivery of care.

## Introduction

Injured pregnant women have a 1.6 times higher risk of death compared with non-pregnant women of childbearing age,^1^ and this cohort accounts for up to 20% of maternal deaths relating to non-obstetric causes.^2–5^ Currently, the incidence of trauma in pregnant women reported in the UK Trauma Audit and Research Network registry is low, with a recorded 5.1% mortality rate.^6^ In the US, data from a study by Mitra *et al* reported that trauma in pregnancy was responsible for 9% of deaths between 1979 and 2017.^4^

Because of the physiological changes experienced by pregnant women and the presence of a fetus, the management of trauma is much more complex, requiring multidisciplinary input from the trauma team, obstetrics and occasionally paediatrics or neonatology if urgent delivery is required to save the life of the mother and the fetus.

Venu *et al* and Tibbott *et al* have provided a structure of how these women should be assessed in the emergency department (ED) on presentation.^5,7^ The classic Advanced Trauma Life Support (ATLS) guidelines of primary survey airways to exposure (A–E) assessment should be followed with some modifications.^8^

To determine how pregnant women with major trauma in England and Wales are assessed and managed, we conducted a survey to identify whether acute hospital trusts had a dedicated protocol or guideline for the management of these patients. The aims of this study were to identify whether management guidelines for pregnant women presenting with major trauma to the ED exist, whether there is consistency across trusts and to extract the key points about the assessment and management of these patients from the guidelines provided. The key points were compared with guidelines/reviews available in the literature for the assessment and management of pregnant patients with major trauma.

## Methods

A freedom of information (FOI) request was emailed to 125 acute NHS trusts in England and six in Wales. Information was requested on trauma protocols or guidelines for pregnant women presenting with trauma to the ED, which specialties were called to attend and whether obstetrics teams were called to attend.

The National Health Service (NHS) trusts had 20 working days to reply to the request. If they had not replied after 20 working days, a reminder was emailed. The initial FOI request was emailed on 6 February 2023 and reminders were emailed out to the NHS trusts until 31 May 2023. If no response was received by 10 June 2023, that trust was classified as a non-responder.

### Statistical analysis

Descriptive statistics were reported for the number of trusts that have a protocol or guideline for pregnant women presenting with trauma to the ED. Descriptive statistics of the number of trusts calling obstetrics and paediatrics in ED were also calculated and reported. A narrative synthesis was used to summarise the key points during initial assessment and secondary survey from the guidelines for those trusts that responded. These were reported along with the percentage of trusts that incorporated each key point in their guideline.

## Results

Of the 131 acute NHS trusts contacted, 126 (96.2%) responded to the FOI request. Twenty-four (19.0%) of the trusts had a dedicated guideline for assessing pregnant women with major trauma presenting to the ED. Ten (7.9%) of the trusts had a generic guideline for assessing pregnant women in the ED, but only six (4.8%) of those included guidance for the management of trauma in pregnant women. Twenty-five (19.8%) trusts had a protocol or algorithm that specified when an obstetric trauma call should be put out by the ED and when a pregnant woman with major trauma should be transferred to a major trauma centre for definitive management, but did not provide any further details about the initial assessment of these women in ED.

Sixty-seven trusts (53.2%) had no specific guideline or protocol for the initial assessment of pregnant women presenting with major trauma to the ED. Four of these trusts provided information for investigations they would consider performing for pregnant women with trauma. Thirty-four (27.0%) trusts had provided information about the assessment or investigations performed in the ED specifically for pregnant women with trauma. Nine of the 34 trusts that provided this information were major trauma centres and 25 were trauma units.

Table 1 summarises the number of trusts that routinely call Obstetrics/gynaecology, Paediatrics or Neonatology to trauma calls for pregnant patients. This is further subdivided into major trauma centres and trauma units.

**Table 1:** Number of trusts that call Obstetrics/Gynaecology, Paediatrics or Neonatology to trauma calls

Additional specialties called in trauma call	Yes	If required	No response
Obstetrics/Gynaecology	88 (69.8)	35 (27.8)	3 (2.4)
Major trauma centres	19 (86.4)	2 (9.1)	1 (4.5)
Trauma units	69 (66.3)	33 (31.7)	2 (1.9)
Paediatrics/Neonatology	46 (36.5)	42 (33.3)	38 (30.2)
Major trauma centres	8 (36.4)	3 (13.6)	11 (50.0)
Trauma units	38 (36.5)	39 (37.5)	27 (26.0)

Values in parentheses are percentages

A narrative summary of the key points outlined in the specific protocols/guidelines are summarised in Tables 2–4. Descriptive statistics on the number of trusts incorporating each of the key points in their protocols or guidelines are summarised in Tables 2–4.

**Table 2:** Summary of the extra considerations provided by the trusts when assessing airway and breathing in pregnant women

Airway/Breathing	No. of trusts (*n* = 34)	Major trauma centres (*n* = 9)	Trauma units (*n* = 25)
Intubation with endotracheal tube to reduce risk of aspiration	7 (20.6)	3 (33.3)	4 (16.0)
Nasogastric tube if intubated to reduce risk of aspiration	6 (17.6)	2 (22.2)	4 (16.0)
Most skilled anaesthetist for RSI if difficult airway	8 (23.5)	3 (33.3)	5 (20.0)
RSI with drugs to be initiated following blood transfusion in hypovolaemic women	6 (17.6)	2 (22.2)	4 (16.0)
Vertical incision for surgical cricothyroidotomy if landmarks not palpable	6 (17.6)	2 (22.2)	4 (16.0)
Adequate preoxygenation with high-flow oxygen because of substantial risk of desaturation	7 (20.6)	3 (33.3)	4 (16.0)
Respiratory alkalosis can be normal in pregnancy with *P*_a_co_2_ < 4kPa, *P*_a_co_2_ > 4 can be a sign of respiratory failure	8 (23.5)	3 (33.3)	5 (20.0)
Chest drains should be inserted in third/fourth intercostal space to reduce risk of diaphragmatic/abdominal injuries	7 (20.6)	3 (33.3)	4 (16.0)

Values in parentheses are percentages

*P*_a_co_2_ = partial pressure of carbon dioxide; RSI = rapid sequence intubation

**Table 3:** Summary of the extra considerations provided by the trusts when assessing circulation, disability and exposure in pregnant women

Circulation/Disability/Exposure	No. of trusts (*n* = 34)	Major trauma centres (*n* = 9)	Trauma units (*n* = 25)
Left lateral tilt or manual displacement of the uterus to the left	18 (52.9)	7 (77.8)	11 (44.0)
Tachycardia and low normal blood pressures suspicious of significant hypovolaemia/Fluid resuscitation and activation of MHP early	11 (32.4)	5 (55.6)	6 (24.0)
Tranexamic acid	16 (47.1)	5 (55.6)	11 (44.0)
Avoid vasopressors (compromise placental perfusion)	6 (17.6)	2 (22.2)	4 (16.0)
Thorough abdominal examination by surgeons and obstetricians (assess tenderness, rigidity, contractions, and fundal height)	14 (41.2)	6 (66.7)	8 (40.0)
Vaginal exam/speculum for evidence of PV bleeding/discharge or spontaneous rupture of membranes	17 (50.0)	7 (77.8)	10 (40.0)
Consider eclampsia as cause of low GCS	8 (23.5)	3 (33.3)	5 (20.0)

Values in parentheses are percentages

GCS = Glasgow Coma Scale; MHP = major haemorrhage protocol; PV = per vaginal

**Table 4:** Summary of the extra considerations provided by the trusts during secondary survey, imaging, and extra investigations for pregnant women

Imaging, Investigations and Secondary survey	No. of trusts (*n* = 34)	Major trauma centres (*n* = 9)	Trauma units (*n* = 25)
Perform clinically indicated CT scans and radiographs	16 (47.1)	5 (55.6)	11 (44.0)
Lead gown can be used to cover the fetus for radiographs	9 (26.5)	4 (44.4)	5 (20.0)
Fetal/Obstetric USS	20 (58.8)	6 (66.7)	14 (56.0)
Fetal monitoring with cardiotocography	25 (73.5)	7 (77.8)	18 (72.0)
Anti-D immunoglobulin prophylaxis to all Rh(−) with major trauma	24 (70.6)	8 (88.9)	16 (64.0)
Kleihauer test to detect fetal cells in the maternal circulation	16 (47.1)	7 (77.8)	9 (36.0)

Values in parentheses are percentages

CT = computed tomography; Rh(−) = Rhesus negative; USS = ultrasound scan

The number of major trauma centres and trauma units that incorporate each of the points in their guideline is shown in Tables 2–4.

## Discussion

Major trauma in pregnant women is rare.^6^ Although uncommon, it is a cohort of patients who require timely and accurate assessment and specific interventions to prevent harm not needed in other injured patient cohorts. Pregnant women need extra considerations during the primary survey and secondary survey because of the changes in maternal physiology and the presence of the fetus. In their reviews, Venu *et al* and Tibbott *et al* summarised the extra considerations and steps required.^5,7^

When establishing whether such steps are commonplace in NHS practice guidelines, we had a high response rate because 96.2% of the NHS trusts replied. Only seven trusts (5.6%) had a guideline that covers the majority of the key points suggested by the ATLS guidelines.^5,7,8^ The percentage of NHS trusts in England and Wales that had a guideline on how to assess a pregnant woman with trauma was low at 27%, with only 19% having guidance specific to major trauma in pregnant women. This highlights the lack of specific guidelines for the assessment and management of this group of patients.

A summary of the key steps that should be followed during the assessment of these patients are discussed further here. We have categorised them in accordance to A–E and ATLS algorithm assessment followed during trauma calls.

### Initial assessment

Additional considerations during the primary survey and secondary survey in pregnant women include modifications for the management of airway, breathing, circulation, disability and exposure.^5,7^ The management of major trauma in pregnancy is a collaborative effort between several specialists. As soon as a pregnant woman with major trauma presents to the ED, a thorough assessment should start. Her vital observations can be used to assess the severity of clinical presentation by using an early obstetric warning system, which is an early warning scoring system adapted to pregnant women because pregnancy accounts for maternal physiological changes.^9,10^ Recognising the severity of critically ill pregnant women at an early stage is crucial to prevent further clinical deterioration.

### Airway and breathing

During pregnancy, there is increased vascularity and oedema of the upper respiratory tract, making it more difficult to achieve a definitive airway in pregnant women.^7,11,12^ Thus, an experienced anaesthetist should be involved in establishing a definitive airway.^7,12^ If the airway is compromised and the woman is hypoxic, rapid sequence intubation (RSI) performed by an experienced anaesthetist should be considered.

Hypocapnia is normal in later pregnancies, hence, normal partial pressure of carbon dioxide (*P*_a_co_2_) may indicate impending respiratory failure.^8^ Because of the increased oxygen requirements during pregnancy and elevation of the diaphragm by the gravid uterus, these women are prone to desaturation.^8^ High-flow oxygen should be provided to pregnant women to maintain their saturation above 95%.^5^ If a chest drain is required in the case of a haemothorax or pneumothorax, it should be placed higher in the third or fourth intercostal space because the gravid uterus can push and elevate the diaphragm.^5,7,8,13^ Pregnant women are at increased risk of aspiration caused by delayed gastric emptying and relaxation of the lower oesophageal sphincter caused by the elevated level of progesterone and the compressing enlarged uterus.^14^ Thus, insertion of a nasogastric tube is advised to decompress the stomach and prevent aspiration.^5,8^

### Circulation, disability and exposure

Plasma volume increases during pregnancy up to 50% and pregnant women can lose 1.2–1.5 litres before showing any signs of blood loss.^8,14^ Therefore, it is important to promptly start fluid resuscitation and activate the major haemorrhage protocol as soon as significant haemorrhage is suspected.^15,16^ Vasopressors should be avoided as much as possible because they reduce the placental circulation.^5,17^

The gravid uterus at more than 20 weeks of gestation can compress the inferior vena cava and aorta causing a reduction in the venous return and leading to hypotension.^14^ Placing pregnant women in the left lateral tilt position or manually displacing the uterus to the left can help relieve the pressure on the inferior vena cava and aorta, and restore the blood pressure.^5,7,8,14^

Teams managing pregnant women with major trauma must ensure that the spine is stabilised using a spinal board before placing the pregnant woman in the left lateral tilt position. Manually moving the uterus to the left is advised if a spinal board is not available.^14^ In case of further clinical deterioration into maternal collapse, left lateral tilt position or manual displacement of the gravid uterus to the left can also improve the efficiency of chest compressions by relieving compression on the inferior vena cava and aorta, improving venous return and cardiac output during resuscitation.^14,18^ It is important to note that these manoeuvres are applicable if a pregnant woman is at more than 20 weeks of gestation, or if the uterus can be palpated above the level of the umbilicus because aortocaval compression is mainly notable starting at 20 weeks of gestation.^14,18^ If cardiopulmonary resuscitation fails within 4min, perimortem caesarean section must be performed within 5min of the onset of maternal collapse to deliver the fetus and relieve the aortocaval compression, thus improving the efficiency of chest compressions.^14,18^

In addition, a thorough abdominal examination should be performed by an obstetrician to assess for traumatic uterine rupture, placental abruption or preterm labour.^5,8^ Anti-D immunoglobulin prophylaxis should be given to all pregnant women with major trauma and a Kleihauer–Betke test should be performed to identify fetal cells in the mother’s circulation.^5,8,19^ A common cause of bleeding in pregnant women is per vaginal bleeding, thus a speculum examination should be performed during primary survey by an obstetrician to assess for bleeding or amniotic fluid.^8^ In case of active haemorrhage, tranexamic acid could be safely administered.^7,20,21^ Finally, eclampsia should be considered as a cause of altered level of consciousness or low Glasgow Coma Scale (GCS) in pregnant women even in the absence of any other warning signs.^8^

### Secondary survey

A computed tomography trauma scan and all the relevant radiographs required to identify injuries in the pregnant woman should be performed because the risk of causing harm to the fetus is minimal.^5,7,22,23^ Widening of the sacro-iliac joints and the pubic symphysis on pelvic radiographs can be normal findings, especially in the later stages of pregnancy and during childbirth.^8,24^ Further investigations should be considered during the secondary survey to assess fetal wellbeing by performing ultrasound scans and cardiotocography.^5,7,8^ The key points for the primary and secondary survey using the ATLS algorithm to assess the pregnant patient with major trauma are summarised in Table 5.

**Table 5:** Summary of the key points that should be assessed when performing a primary and secondary survey in pregnant women

Airway	Breathing	Circulation	Disability	Exposure	Secondary survey
Intubation with endotracheal tube to reduce risk of aspiration Intubation should be performed by an experienced anaesthetist and RSI should be considered if the airway is compromised Nasogastric tube should be inserted to reduce the risk of aspiration	Normal *P*_a_co_2_ can be a sign of impending respiratory failure High-flow oxygen to maintain saturations Chest drain should be inserted in third/fourth intercostal space to reduce risk of diaphragmatic/abdominal injuries	Left lateral tilt or manual displacement of the uterus to the left Tachycardia and low normal blood pressures are suspicious of significant hypovolaemia Give tranexamic acid early if there is significant haemorrhage MHP should be initiated early if significant haemorrhage is suspected Avoid vasopressors if possible	Consider eclampsia as cause of low GCS	Thorough abdominal examination by surgeons and obstetricians Vaginal exam/speculum for evidence of PV bleeding/discharge or spontaneous rupture of membranes	Perform clinically indicated CT scans and x rays Fetal/Obstetric USS Fetal monitoring with cardiotocography Anti-D immunoglobulin prophylaxis to all Rh(−) with major trauma

CT = computed tomography; GCS = Glasgow Coma Scale; MHP = major haemorrhage protocol; *P*_a_co_2_ = partial pressure of carbon dioxide; PV = per vaginal; Rh(−) = Rhesus negative; RSI = rapid sequence intubation; USS = ultrasound scan

In summary, only 19% of trusts had a guideline for assessing pregnant women with major trauma presenting to the ED and a smaller percentage addressed the specifics identified in previous works. Therefore, there is a need to produce agreed unified national guidance for better managing pregnant women with major trauma, aiming to improve maternal health outcomes and prevent future maternal morbidity and mortality.

### Study limitations

This is a survey of the guidelines for each trust and might not necessarily capture the actual practice that occurs during an obstetric trauma call in each hospital. A prospective study auditing the steps involved in obstetric trauma calls in each trust might be more informative of current practice. In addition, smaller hospitals might not be as familiar in dealing with major trauma in pregnant women compared with major trauma centres, and thus not have developed guidelines for pregnant women.

## Conclusion

Pregnant women with major trauma are a complex group to manage given the changes in maternal physiology and the presence of the fetus. The heterogeneity of guidelines available across the different trusts necessitates the development of a national guideline for the assessment of these women to improve delivery of care.
